# Using Item Response Theory for Explainable Machine Learning in Predicting Mortality in the Intensive Care Unit: Case-Based Approach

**DOI:** 10.2196/20268

**Published:** 2020-09-25

**Authors:** Adrienne Kline, Theresa Kline, Zahra Shakeri Hossein Abad, Joon Lee

**Affiliations:** 1 Department of Biomedical Engineering University of Calgary Calgary, AB Canada; 2 Undergraduate Medical Education Cumming School of Medicine University of Calgary Calgary, AB Canada; 3 Data Intelligence for Health Lab Cumming School of Medicine University of Calgary Calgary, AB Canada; 4 Department of Psychology University of Calgary Calgary, AB Canada; 5 Department of Community Health Sciences Cumming School of Medicine University of Calgary Calgary, AB Canada; 6 Department of Cardiac Sciences Cumming School of Medicine University of Calgary Calgary, AB Canada

**Keywords:** item response theory, machine learning, statistical model, mortality

## Abstract

**Background:**

Supervised machine learning (ML) is being featured in the health care literature with study results frequently reported using metrics such as accuracy, sensitivity, specificity, recall, or F1 score. Although each metric provides a different perspective on the performance, they remain to be overall measures for the whole sample, discounting the uniqueness of each case or patient. Intuitively, we know that all cases are not equal, but the present evaluative approaches do not take case difficulty into account.

**Objective:**

A more case-based, comprehensive approach is warranted to assess supervised ML outcomes and forms the rationale for this study. This study aims to demonstrate how the item response theory (IRT) can be used to stratify the data based on how *difficult* each case is to classify, independent of the outcome measure of interest (eg, accuracy). This stratification allows the evaluation of ML classifiers to take the form of a distribution rather than a single scalar value.

**Methods:**

Two large, public intensive care unit data sets, Medical Information Mart for Intensive Care III and electronic intensive care unit, were used to showcase this method in predicting mortality. For each data set, a balanced sample (n=8078 and n=21,940, respectively) and an imbalanced sample (n=12,117 and n=32,910, respectively) were drawn. A 2-parameter logistic model was used to provide scores for each case. Several ML algorithms were used in the demonstration to classify cases based on their health-related features: logistic regression, linear discriminant analysis, K-nearest neighbors, decision tree, naive Bayes, and a neural network. Generalized linear mixed model analyses were used to assess the effects of case difficulty strata, ML algorithm, and the interaction between them in predicting accuracy.

**Results:**

The results showed significant effects (*P*<.001) for case difficulty strata, ML algorithm, and their interaction in predicting accuracy and illustrated that all classifiers performed better with easier-to-classify cases and that overall the neural network performed best. Significant interactions suggest that cases that fall in the most arduous strata should be handled by logistic regression, linear discriminant analysis, decision tree, or neural network but not by naive Bayes or K-nearest neighbors. Conventional metrics for ML classification have been reported for methodological comparison.

**Conclusions:**

This demonstration shows that using the IRT is a viable method for understanding the data that are provided to ML algorithms, independent of outcome measures, and highlights how well classifiers differentiate cases of varying difficulty. This method explains which features are indicative of healthy states and why. It enables end users to tailor the classifier that is appropriate to the difficulty level of the patient for personalized medicine.

## Introduction

### Background

This study aims to demonstrate an approach to assess the effectiveness of binary machine learning (ML) classification, which is an alternative to the more traditional single scalar measures in the literature. Our approach uses an item response theory (IRT) model to enhance the understanding of the data set on which ML protocols are run as well as the results of the classification outcomes. Aspects of IRT’s utility have recently surfaced in the ML literature, including comparisons of collaborative filtering [1], evaluation of natural language processing systems [2], identification of initial computer adaptive learning items [3], assessment of the utility of ML classifiers [4], and ML feature selection [5]. However, using IRT in the manner proposed in this study has not yet been undertaken.

The varied and numerous contexts (eg, business, finances, medicine, home, government agencies) in which ML is being used is no less than staggering [6]. Since the advent of the development of ML classification protocols, there has been a commensurate interest in assessing and comparing their efficacy [7]. Evaluation techniques fall into several major categories [8-12]. Many techniques use estimates of single scalar values to summarize the quality of classification based on the frequencies in a confusion matrix (true-positive, false-positive, true-negative, and false-negative). The most common measures include accuracy, precision, negative predictive value, sensitivity, and specificity, although some combine sensitivity and specificity (eg, Youden index, likelihoods, and discriminant power) [13]. Further refinements of scalar estimates have been introduced including reducing the amount of bias and variance of the estimate that have enhanced their interpretability [14], presenting statistical comparisons (eg, *t* test, conservative z, McNemar test) between ML protocol scalar outcomes [15] and assessing the invariance of estimates with changes in the confusion matrix [16]. Graphical presentations of the confusion matrix data at various points along a continuum include gain and lift charts, receiver operating characteristic curves, and area under the curve (AUC). These provide a more comprehensive depiction of the various scalar measures [12] by contextualizing them.

Despite the advances in metric development, there is an interest in developing more extensive descriptions of ML classification outcomes. For example, it has been argued that “... any single scalar measure has significant limitations” and “that such measures ... oversimplify complex questions and combine things that should be kept separate” [17]. Another issue is that many different programs, search and optimize strategies, and evaluation approaches have populated the literature, sparking researchers to attempt to systematize the findings for more general consumption [18]. Some reviews have supported the contention that ML algorithms should outperform human experts [19], but others have found that overly complex approaches used in some ML models are no better than simpler, more intuitive models [20]. Studies should give readers an understanding of the reasons why algorithms perform differently, rather than simply providing results of differences between 2 scalar summary values [21,22].

These comments are consistent with general calls for a fuller explanation regarding the interpretability of ML studies [23], particularly in the biomedical and health fields that have been slower to exploit this technology. Overreliance on text-based information rather than contextual elements and the inherent uncertainty in medical decision making have been cited as problems in applying ML findings [24]. The associations within data sets that are theory-free offer little guidance with regard to improving clinical care [25]. Medical professionals are not well trained in how ML works and thus are not able to critically evaluate the utility of the reported findings [26].

The ML criticism of the lack of attention to the unique characteristics of the individual cases is the focus of this study. We propose to address this challenge using a more comprehensive, case-nuanced approach. Although there has been some work in this regard, such as the now accepted wisdom that standard classifiers do not work well with imbalanced data [27], a focus on the individual cases that fall into the *miss* or *false-positive* categories has only rarely been investigated as a point of interest [28].

This lack of attention is highlighted in the assessment study of various ML models; they often result in comparable outcomes as similar percentages of cases are misclassified regardless of the model used [29]. Some insight into this phenomenon was brought to light in a study on benchmarking data sets, where some sets had more *difficult*-to-classify cases and other sets contained largely *easy*-to-classify cases, providing similar results at the aggregate level regardless of the ML approach used [30]. It has been argued that cleaning up the data by eliminating some cases in the training data set is an appropriate tactic to improve classification accuracy [31]. We disagree with this approach and instead argue that hard-to-classify cases should be examined in a more systematic manner. This study shifts the focus from the level of utility of an ML only at the aggregate sample level toward pinpointing where that model falls short and, more importantly, why the model falls short. The process described identifies, *a priori*, which cases in the data set (balanced or imbalanced) will be more or less difficult to classify. The evaluation of ML algorithms across these cases will help to understand why these cases are difficult to classify. Examining this phenomenon in detail is as important as the classification accuracy index of the data set as a whole.

There are 2 fundamental building blocks to any ML system: the features of interest and the cases in the data set. To investigate the research question in this study, methods derived from IRT were employed as they simultaneously estimate the characteristics of both features and cases. Understanding this phenomenon allows medical professionals to tailor the classifier to the patient.

### Similarities Between ML and Test Taking

Following an examination, there are discussions by students about the test items; often they remark “that question was hard, what did you put?” or “that was as easy question.” Such comments reflect the purposeful construction of the test items. Some items are designed to be relatively easy to pass whereas others are designed to be more difficult such that only a few can pass. Similarly, students talk about the test takers—“she always gets the highest score in the class” or “I think I have a 50-50 chance of passing.” Test takers are quite cognizant of the fact that not all test items are created equal and that not all test takers have the same ability. These fundamental assumptions give rise to IRT, where the characteristics of the items and of the students are modeled together, providing a clearer picture about which items discriminate between which test takers.

A parallel can be drawn between a set of students passing or failing a test based on their performance on a set of items with a set of patients being classified into 1 of 2 categories (alive or not alive) based on their scores on a set of health-related features. Using the *test* item information, an ML classifier should be able to predict which students will pass or fail the test, and using the feature information, an ML classifier should be able to predict which patients will be alive or not alive. There is likely to be a base level at which it correctly classifies cases as belonging to 1 group or another by chance alone, and additional case information on each feature should enhance the prediction. Some cases can be easily partitioned into the *pass the test* (ie, they pass all the items) or *fail the test* (ie, they fail all the items). Cases with more moderate levels of mastery however would be expected to pass some and fail some items (features in ML terms). It is these *difficult* cases where classifiers would be less likely to successfully predict their probability of passing or failing the test. One option to enhance prediction is to add more features to help classify these more difficult cases, but doing so results in high dimensionality, overfitting models, difficult-to-interpret findings, and nongeneralizing results. This quandary is a classic optimization problem in the ML literature.

As not all test takers score 100% or 0% on an examination, some combination of right and wrong answers to questions provides an index of individual test-taker ability in completing the test. The term ability (symbolized by the term theta, θ) is used in the psychometric literature where IRT evolved and is used to describe any latent construct of interest being measured. In this study, within-range or out-of-range laboratory values and vital signs as well as demographic information comprise the features in our data sets. Thus, we can ascertain a case’s placement with respect to the underlying distribution of *unhealthiness*. These individual case-based indices create a distribution of unhealthiness across all features (or in our case laboratory values, vital signs, and demographic information). Depending on where individual patients fall on the distribution, the ease with which ML classifiers correctly predict the outcome (mortality) is expected to be affected—those concentrated in the central area of the distribution will be more challenging to correctly classify relative to those cases lying more at the tails of the distribution. Thus, rather than labeling case scores as being on a healthy-unhealthy continuum, suggesting these scores might only be useful in a health context, we use the classification difficulty index (CDI) because of their ease or difficulty in being able to be correctly classified using supervised ML.

The process of generating CDIs on the unhealthiness continuum will be carried out without using the outcome variable of mortality itself, that is, IRT provides case-based scores (CDIs) that can be examined before the data as a collective is subjected to an ML protocol.

### Specific Study Hypotheses and Research Questions

The IRT analysis provides case-based CDIs using a set of feature characteristics that do not use the information on the outcome classification variable. CDIs for the sample are generated along the normal distribution, with a mean 0.0 (SD 1.0). It is hypothesized that cases with more centrally located CDIs will be less likely to be classified correctly, whereas cases with more peripherally located CDIs will be more likely to be classified correctly. One research question is as follows: Will some ML classifiers be more accurate in classifying cases at all CDIs? Another research question is as follows: Will some ML classifiers be more accurate than others in classifying cases at different CDIs? Identifying these cases *a priori* provides an alternative manner to evaluate different ML protocols or classification methods and will advance our understanding of ML findings and the data they are being fed with.

## Methods

### Data Sets

Data were obtained through 2 large, freely available data sets. One was the MIMIC-III (Medical Information Mart for Intensive Care III) database housing health data of >40,000 critical care unit patients at the Beth Israel Deaconess Medical Center admitted between 2001 and 2012 [32,33]. The other was the electronic intensive care unit (eICU) Collaborative Research Database that houses data from critical care unit patients from across the continental United States admitted between 2014 and 2015 [34].

### Case Inclusion

Databases were queried using the SQL plug-in for Python (Python Software Foundation). Case inclusion criteria were as follows: (1) age 16 years, (2) at least three-fourth of the features of interest were available for a select case (patient), leading to subsequent imputation, and (3) first hospital visit in the case of repeated patients. Features of predictive interest were selected based on 2 common severity of illness scores: Simplified Acute Physiology Score II and Acute Physiology and Chronic Health Evaluation IV for MIMIC-III and eICU, respectively. To test the hypothesis with both balanced and imbalanced data sets, the number of *death* cases in both data sets (coded *1*) was noted and the same number of cases of *no death* was then randomly selected and incorporated into the balanced data sets. Imbalanced data sets were created by randomly sampling twice as many *no death* cases compared with *death* cases. We used the 1/3:2/3 imbalance ratio to detect any change in results using a somewhat mildly imbalanced than an extremely imbalanced set.

For the MIMIC-III data set, there were 4039 cases that experienced *death in hospital*, resulting in a final and balanced sample size of 8078 and imbalanced sample size of 12,117. In the eICU data set, there were 10,970 *death in hospital* cases. Employing the same methodology resulted in a balanced sample size of 21,940 and an imbalanced sample size of 32,910.

### Features

The features included demographic, procedural, pre-existing conditions, and laboratory values (Tables 1 and 2). Normal values were presented and were obtained from the Medical Council of Canada [35] unless otherwise noted. Laboratory values represent the worst values taken during the intensive care unit (ICU) stay in both data sets in the first 24 hours. In the IRT component of the analyses, variables were dichotomized into disease-promoting states (1) akin to *failing* the item on the test or disease-protective states (0; passing the item). Values that fell outside the normal laboratory ranges were coded as 1 (too low or too high). Pre-existing conditions were coded as 1 (present) or 0 (absent). Age was demarcated at 65 years, with those aged >65 years coded 1 and those aged ≤65 years coded 0 [36-39]. For the sex variable, men were assigned 1 and women were assigned 0 [40]. Imputation of missing data was performed using a multiple imputation chained equations technique using the *impyute* library in Python 3.7.7 to preserve the pre-existing distribution of features.

**Table 1:** Medical Information Mart for Intensive Care III variables based on Simplified Acute Physiology Score II.

Feature name	Description	Normal values, units
AIDS	Pre-existing diagnosis	Absent: 0, 0 or 1
Heme malignancy	Pre-existing diagnosis	Absent: 0, 0 or 1
Metastatic cancer	Pre-existing diagnosis	Absent: 0, 0 or 1
Minimum GCS^a^	Glasgow Coma Scale	15^b^, 1-15
WBC^c^ minimum	Lowest white blood cell	4-10, 10^9^
WBC maximum	Highest white blood cell	4-10, 10^9^
Na minimum	Sodium minimum	135-145, mmol/L
Na maximum	Sodium maximum	135-145, mmol/L
K minimum	Potassium minimum	3.5-5, mmol/L
K maximum	Potassium maximum	3.5-5, mmol/L
Bilirubin maximum	Bilirubin maximum	≤1.52, mg/dL
HCO_3_ minimum	Bicarbonate minimum	24-30, mmol/L
HCO_3_ maximum	Bicarbonate maximum	24-30, mmol/L
BUN^d^ minimum	Blood urea nitrogen minimum	7-22, mg/dL
BUN maximum	Blood urea nitrogen maximum	7-22, mg/dL
PO_2_	Partial pressure of oxygen	85-105, mm Hg
FiO_2_	Fraction of inspired oxygen	21, %
Heart rate mean	Mean heart rate	60-100, bpm
BP mean	Mean systolic blood pressure	95-145, mm Hg
Max temp	Maximum temperature	36.5-37.5, ℃
Urine output	Urine output	800-2000^e^, mL/24h
Sex	Male or female	Male: 1, Female: 0, Male or female
Age	Age in years	≤65: 0, years
Admission type	Emergency or elective	Emergency: 1; else: 0, N/A^f^

^a^GCA: Glasgow Coma Scale.

^b^Teasdale and Jennett, 1974 [41]; Teasdale and Jennett, 1976 [42].

^c^WBC: white blood cell.

^d^BUN: blood urea nitrogen.

^e^Medical CMP, 2011 [43].

^f^N/A: not applicable.

**Table 2:** Electronic intensive care unit data set variables based on Acute Physiology and Chronic Health Evaluation IV.

Feature name	Description	Normal values, Units
GCS^a^	Glasgow Coma Scale	15^b^, 1-15
Urine output	Urine output in 24 hours	800-2000^c^, mL/24 hour
WBC^d^	White blood cell count	4-10, 10^9^
Na	Serum sodium	135-145, mmol/L
Temperature	Temperature in Celsius	36.5-37.5^e^, ℃
Respiration rate	Highest white blood cell	12-20^f^, breaths/min
Heart rate	Heart rate/min	60-100^f^, bpm
Mean blood pressure	Mean arterial pressure	70-100^g^, mm Hg
Creatinine	Serum creatinine	0.57-1.02 (F^h^); 0.79-1.36 (M^i^), mEq/L
pH	Arterial pH	7.35-7.45, N/A^j^
Hematocrit	Red blood cell volume	37-46 (F); 38-50 (M), %
Albumin	Serum albumin	3.5-5.0, g/dL
PO_2_	Partial pressure of oxygen	85-105, mm Hg
PCO_2_	Partial pressure carbon dioxide	35-45, mm Hg
BUN^k^	Blood urea nitrogen maximum	7-22, mg/dL
Glucose	Blood sugar level	68-200, mL/dL
Bili	Serum bilirubin	≤1.52, md/dL
FiO_2_	Fraction of inspired oxygen	21^l^, %
Sex	Male or female	Male: 1; female: 0, M or F
Age	Age in years	≤65: 0, years
Leukemia	Pre-existing diagnosis	Absent: 0, 0 or 1
Lymphoma	Pre-existing diagnosis	Absent: 0, 0 or 1
Cirrhosis	Pre-existing diagnosis	Absent: 0, 0 or 1
Hepatic failure	Pre-existing diagnosis	Absent: 0, 0 or 1
Metastatic cancer	Pre-existing diagnosis	Absent: 0, 0 or 1
AIDS	Pre-existing diagnosis	Absent: 0, 0 or 1
Thrombolytics	Medical intervention	Absent: 0, 0 or 1
Ventilator	Medical intervention	Absent: 0, 0 or 1
Dialysis	Medical intervention	Absent: 0, 0 or 1
Immunosuppressed	Medical intervention	Absent: 0, 0 or 1
Elective surgery	Medical intervention	Absent: 0, 0 or 1

^a^GCS: Glasgow Coma Scale.

^b^Teasdale and Jennett, 1974 [41]; Teasdale and Jennett, 1976 [42].

^c^Medical CMP, 2011 [43].

^d^WBC: white blood cell.

^e^Lapum et al. 2018 [44].

^f^MDCalc [45].

^g^Healthline [46].

^h^F: female.

^i^M: male.

^j^N/A: not applicable.

^k^BUN: blood urea nitrogen.

^l^eICU Collaborative Research Database [47].

### IRT Analyses

Using the IRTPRO (Scientific Software International) program, a 2-parameter logistic model (2PL) was run on the dichotomous data. The program uses a marginal maximum likelihood estimation procedure to calculate feature and case parameters [48] and assumes that respondents belong to a population that can be characterized by their placement on a latent normal probability distribution—*unhealthiness* in this study with the left and right sides of the distribution indicating better and worse health, respectively [49]. Although higher scores on the latent distribution in IRT usually indicate better outcomes (eg, students have passed more items on a test), in this context, higher scores mean more of the patients’ features were out of range and are thus associated with worse outcomes (ie, higher likelihood of death). The output generates logistic item characteristic curves that describe each feature’s relationship to the underlying distribution. For each feature, 2 characteristics were estimated, slope and location.

**Figure 1:**
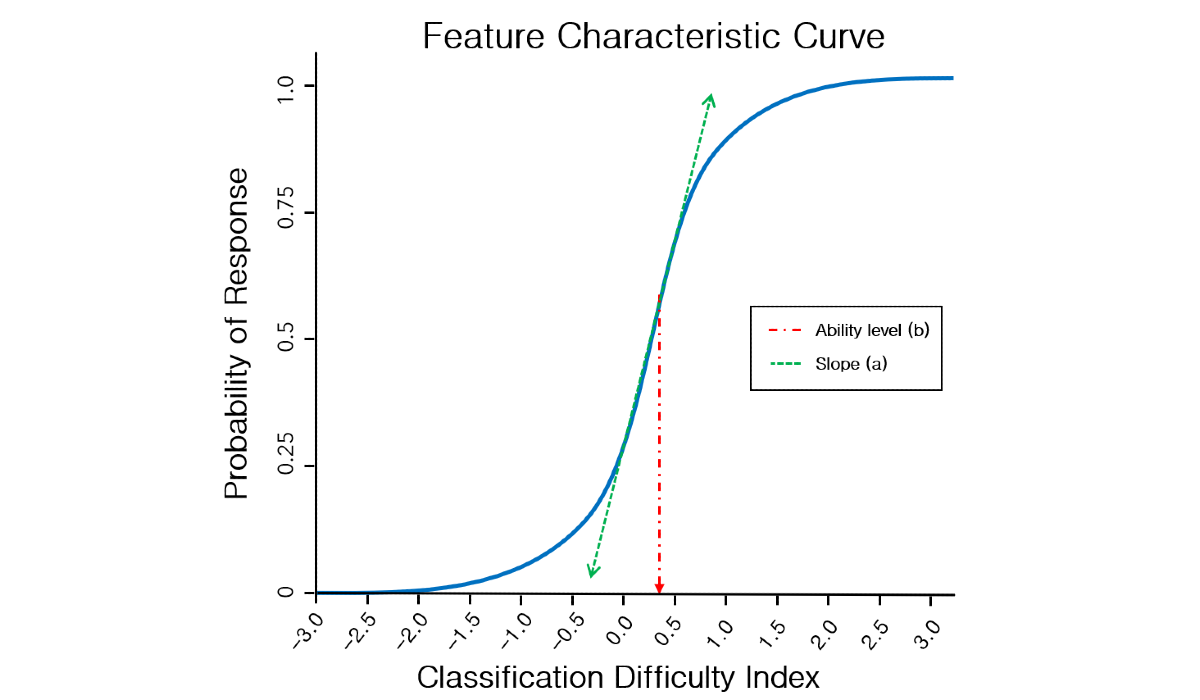
Characteristic curve using a 2-parameter logistic model.

Equation 1 shows a 2PL model in IRT; slope (a_i_) captures the *discriminability* capacity of the feature. Feature functions with flat slopes indicate that they are not very discriminatory, whereas those with steep slopes are highly discriminatory, particularly at the inflection point. The location (b_i_) denotes where along the function the inflection point occurs. As the functions are set along the standard normal distribution (mean 0.0, SD 1.0), this point indicates where along the unhealthiness continuum the feature is most likely to differentiate cases. An example is presented in Figure 1.

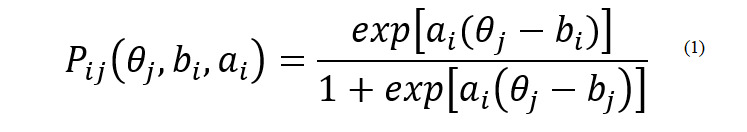



CDI estimation in a 2PL model is calculated based on equation 2, where the probability of obtaining the correct answer is based on the scores on the items’ u_i_ weighted by a_i_.

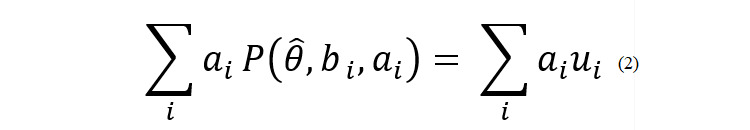

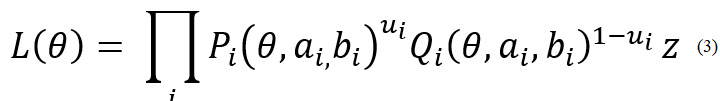





Equation 3, where u_i_ ∈ (0, 1) is the score on item i, is called the likelihood function. It is the probability of a response pattern given the CDIs and the item parameters across cases. There is 1 likelihood function for each response pattern, and the sum of all such functions equals 1 at any value of the distribution. On the basis of the pattern of each case’s values on the features, the program uses a Bayesian estimation process that provides a CDI on the unhealthiness continuum for each case in the data set.

CDIs are reported on the standard normal distribution and typically range between −2.50 and +2.50. Each case’s CDI has its own individual SE around it based on the individual’s pattern of results across all features and their unique characteristics. Using the results from the 2PL model, it was possible to identify which of the cases were more centrally or more peripherally located on the distribution and thus would be less or more likely to be accurately classified into their respective categories (no death or death).

To allow for easy visualization and testing of effects, several strata bins were created into which continuous IRT CDIs could be assigned. These *bins* were separated at every 0.5 difficulty change in the distribution of the data. The first bin was centered over the *0.0* mark to denote the most difficult cases and subsequent bins were demarcated at 0.5 levels toward each periphery. This process of bin allocation continued until all observed CDIs for the cases were accounted for.

### ML Analyses

Multiple ML algorithms were tested using the original feature values for both MIMIC-III and eICU data sets. These included logistic regression, linear discriminant analysis, K-nearest neighbors, decision tree, naive Bayes, and neural network. Both the K-nearest neighbors and neural network had their hyperparameters optimized by a grid search. In the case of the K-nearest neighbors, the search grid included K from 1 to 40 and distance methods of Minkowski, Hamming, and Manhattan. The grid investigated for the neural network included activation functions such as softmax, softplus, softsign, relu, Tanh, sigmoid, and hard sigmoid; learning rates such as 0.001, 0.01, 0.1, 0.2, and 0.3; and hidden neurons in a single hidden layer of 1, 5, 10, 15, 20, 25, and 30. In each of these methods, a 10-fold cross-validation was performed, and the numerical prediction was extracted for each case and then reassociated with its subject ID number for graphical plotting. The evaluation methods, accuracy, precision, recall, F1, and AUC metrics were calculated. Accuracy was used to assess the hypotheses and research questions.

### Comparison Analyses

To test the main effects of CDI and the repeated measure of the ML classifier as well as their interaction on each case’s accuracy score (0,1), generalized linear mixed model (GLMM) [50] analyses were conducted using the GENLINMIX program of SPSS 23 [51]. GENLINMIX uses the penalized quasi-likelihood estimation method for fixed effects. Separate analyses for each of the balanced and imbalanced data sets were conducted. The standard form of the GLMM is shown in equations 4 and 5. y is a response vector, and b is the random effects vector. *Distr* is a conditional distribution of y given b. µ is the conditional mean, and is the dispersion parameter.

In equation 5, g(µ) is the logit link function that defines the relationship between the mean response µ and the linear combination of predictors. X represents the fixed effects matrix, and Z is a random effects matrix, where is simply an offset to the model.

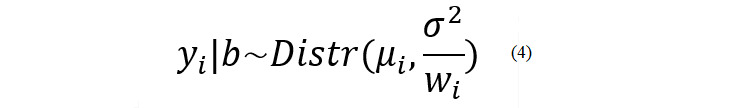

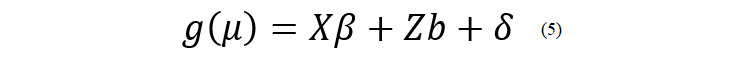





The models specified that (1) all effects are fixed, (2) the dependent variable follows a binomial distribution, and thus the predictors and criterion are linked via a logit function, (3) the residual covariance matrix for the repeated measure (ML classifier) is diagonal, and (4) the reference category was set to 0. Follow-up paired-comparison tests on the estimated marginal and cell means used a *P* level of<.001 to protect against a type I error.

## Results

### IRT 2PL Model Results

**Figure 2:**
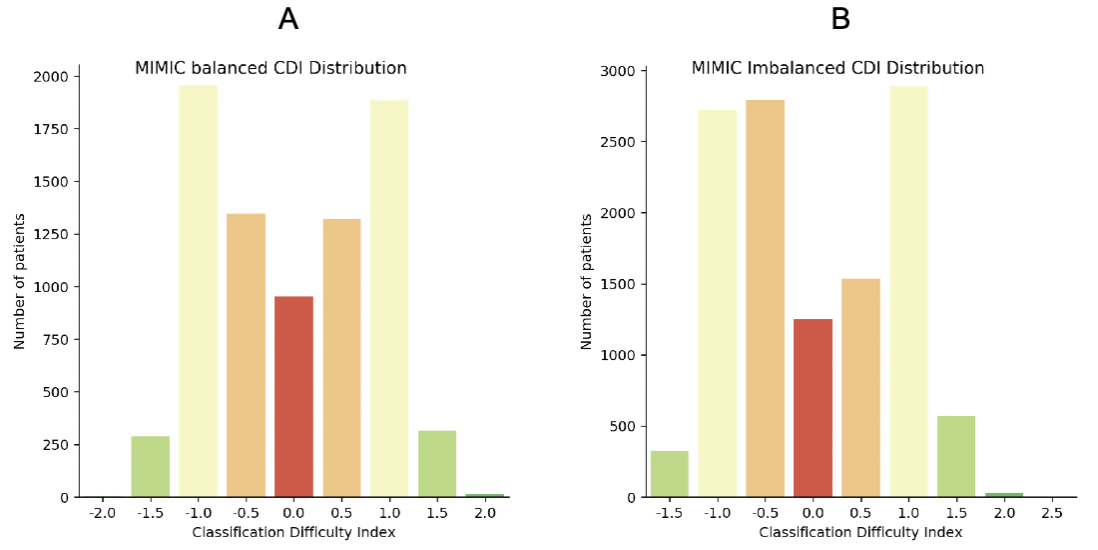
Classification Difficulty Indexes in MIMIC-III (A) balanced and (B) imbalanced data. CDI: classification difficulty index; MIMIC: Medical Information Mart for Intensive Care.

**Figure 3:**
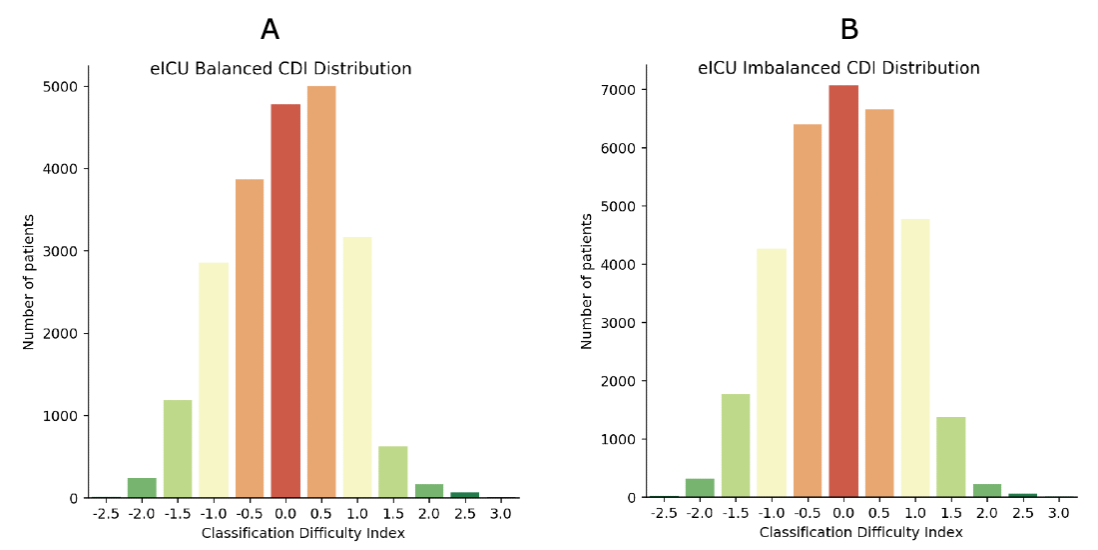
Classification Difficulty Indexes in eICU (A) balanced and (B) imbalanced data. eICU: electronic Intensive Care Unit; DT: decision tree; KNN: K-nearest neighbors; LDA: linear discriminant analysis; LR: logistic regression; NB: naive Bayes; NN: neural network.

**Figure 4:**
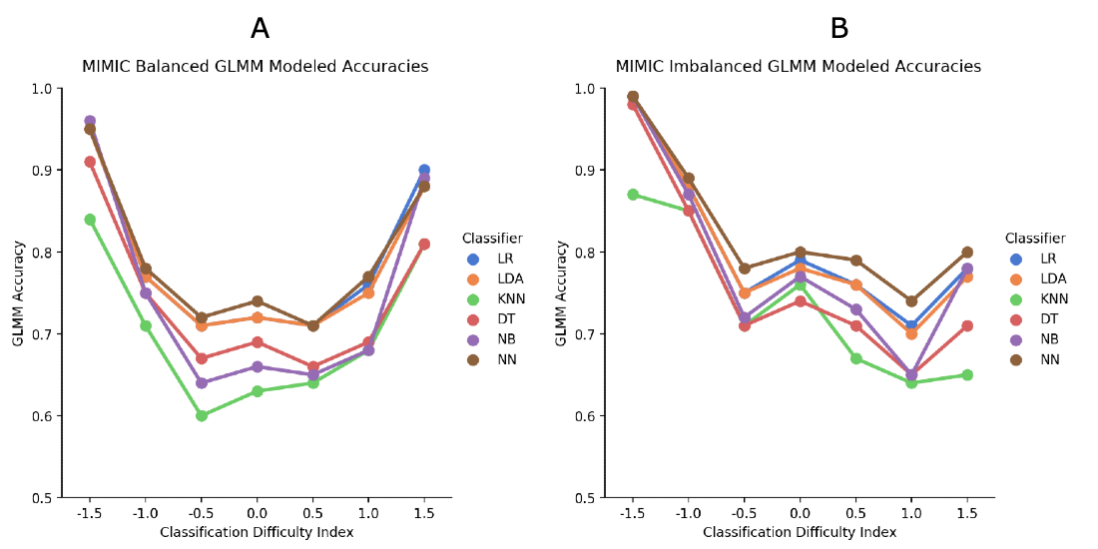
Medical Information Mart for Intensive Care (MIMIC) III generalized linear mixed model (GLMM) accuracy results; machine learning classifier against CDI for (A) balanced and (B) imbalanced data. DT: decision tree; KNN: K-nearest neighbors; LDA: linear discriminant analysis; LR: logistic regression; NB: naive Bayes; NN: neural network.

**Figure 5:**
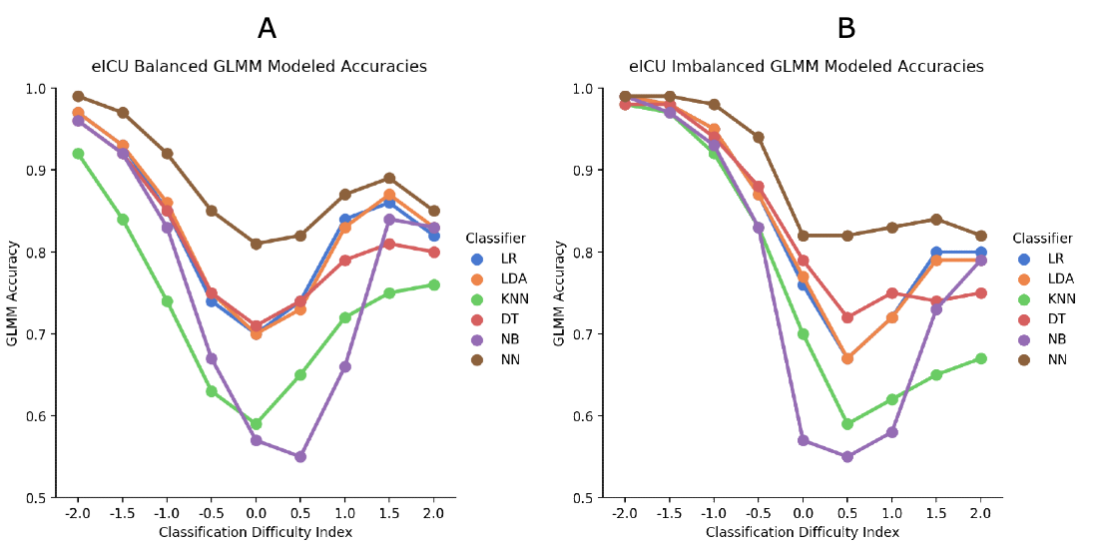
Electronic intensive care unit (eICU) generalized linear mixed model (GLMM) accuracy results; machine learning classifier against CDI for (A) balanced and (B) imbalanced data. DT: decision tree; KNN: K-nearest neighbors; LDA: linear discriminant analysis; LR: logistic regression; NB: naive Bayes; NN: neural network.

Descriptive results of case CDIs are shown in Table 3, and frequency distributions are shown in Figures 2 and 3 (MIMIC-III) and Figures 4 and 5 (eICU).

It should be noted that the 2 data sets have different distributions, and this fingerprint is inherently unique to the data set processed.

**Table 3:** Item response theory case classification difficulty index results.

Data set	CDI^a^ range	Overall, mean (SD)	Point-biserial correlations^b^		No death, mean (SD)	Death, mean (SD)	*Two-tailed t* value^c^	
			*r* value	*P* value			*t* test (df)	*P* value
MIMIC-III^d^ balanced	−1.81 to +2.16	0.00 (0.85)	0.37	<.001	−0.32 (0.79)	0.32 (0.80)	35.76 (8077)	<.001
MIMIC-III imbalanced	−1.70 to +2.27	0.00 (0.85)	0.35	<.001	−0.21 (0.80)	0.42 (0.80)	40.88 (12116)	<.001
eICU^e^ balanced	−2.63 to +2.83	0.00 (0.80)	0.50	<.001	−0.40 (0.73)	0.40 (0.64)	86.18 (21939)	<.001
eICU imbalanced	−2.55 to +2.93	0.00 (0.81)	0.51	<.001	−0.29 (0.73)	0.59 (0.61)	109.09 (32909)	<.001

^a^CDI: classification difficulty index.

^b^Between CDI and outcome (no death or death).

^c^Difference between no death and death means.

^d^MIMIC III: Medical Information Mart for Intensive Care.

^e^eICU: electronic intensive care unit.

Using the feature parameter estimates and case CDI, the unique differentiating capacity for each feature can be depicted by calculating the probability of each case falling into the 0 (no death) or 1 (death) categories. For example, the slope and location parameters for the blood urea nitrogen (BUN) minimum and urine output for the 2 MIMIC-III data sets are shown in Table 4. The higher slope of the BUN minimum feature is contrasted with the very low slope of the urine output feature. These differences highlight the importance of some features over others in terms of being useful in categorizing cases.

**Table 4:** Medical Information Mart for Intensive Care III feature parameters.

Feature parameters		Slope	Location
**Balanced**			
	Blood urea nitrogen (minimum)	5.64	0.09
	Urine output	0.15	−2.23
**Imbalanced**			
	Blood urea nitrogen (minimum)	5.22	0.02
	Urine output	0.09	−3.59

Similar to the MIMIC-III results, the IRT analyses of the eICU showed that BUN was a highly discriminating feature whereas urine output was not (Table 5). In fact, many of the features for both MIMIC-III and eICU were not discriminatory (slopes of <0.35 [52]).

**Table 5:** Electronic intensive care unit feature parameters.

Feature parameter		Slope	Location
**Balanced**			
	Blood urea nitrogen (minimum)	1.55	−0.33
	Urine output	0.04	−1.19
**Imbalanced**			
	Blood urea nitrogen (minimum)	1.49	−0.1
	Urine output	0.03	−1.39

### ML Classification Results

Checking the K-nearest neighbors grid warranted using Manhattan distancing and 27 nearest neighbors for MIMIC-III and Manhattan distancing with 19 neighbors for eICU. The neural network grid search results returned an optimum learning rate of 0.001, activation function softmax, and a number of hidden nodes, 15 for MIMIC-III and 17 for eICU.

Traditional metrics of accuracy, precision, recall, F1, and AUC are presented for MIMIC-III in Table 6.

**Table 6:** Medical Information Mart for Intensive Care III classification performance in traditional metrics.

Metric		LR^a^ (%)	LDA^b^ (%)	KNN^c^ (%)	DT^d^ (%)	NB^e^ (%)	NN^f^ (%)
**Balanced**							
	Accuracy	75.3	75.0	67.2	70.9	70.4	76.1
	Precision	75.8	75.6	69.3	71.1	79.5	75.6
	Recall	74.3	73.8	61.8	70.6	54.9	77.2
	F1	75.0	74.7	65.3	70.8	64.9	76.4
	AUC^g^	75.3	75.0	67.2	70.9	70.4	76.5
**Imbalanced**							
	Accuracy	78.3	77.9	72.8	73.7	75.3	80.5
	Precision	73.3	73.8	63.1	60.6	67.7	72.7
	Recall	54.8	52.1	44.4	60.6	49.6	66.6
	F1	62.7	61.1	52.2	60.6	57.3	69.5
	AUC	72.4	71.4	65.7	70.9	68.9	76.9

^a^LR: logistic regression.

^b^LDA: linear discriminant analysis.

^c^KNN: K-nearest neighbor.

^d^DT: decision tree.

^e^NB: naive Bayes.

^f^NN: neural network.

^g^AUC: area under the curve.

In both the balanced and imbalanced MIMIC-III data sets, the neural network outperformed the other classifiers (balanced: accuracy was 76.1% and imbalanced: accuracy was 80.5%) using traditional metrics. It is worth highlighting the role an imbalanced data set has on an increased accuracy and a reduction in precision, recall, and F1.

Table 7 shows our proposed method of demonstrating accuracy as a function of CDI. The metric used in Table 7 is accuracy as F1, recall, and precision were undefined in the extreme negative (where features were predominantly 0), and no cases of *death* existed by which to divide. A parabolic relationship existed in the accuracy level and the strata values, where those more distant from the strata bin=0 were more likely to be classified correctly. ML researchers should be most interested in the *problematic* cases CDI bin 0.0 and where we observe that all classifiers struggle with prediction. These results suggest that even if a classifier outperforms its counterparts as shown in the traditional metrics of Table 6 (eg, neural network), it may be surpassed in the more fine-grained approach shown in Table 7 (eg, naive Bayes algorithm within the +1.5 CDI bin of the balanced data set).

In both the balanced and the imbalanced eICU data sets (Table 8), the neural network outperformed the other classifiers using traditional metrics. Similar to the MIMIC-III findings, the imbalanced data set resulted in increased accuracy and decreased precision, recall, and F1.

Table 9 shows our alternative method of demonstrating accuracy as a function of CDI. Cases that were more distant from the strata bin=0 were more likely to be classified correctly.

**Table 7:** Item response theory–based Medical Information Mart for Intensive Care III mortality prediction accuracy stratified by classification difficulty index.

Number of cases		CDI^a^	LR^b^ (%)	LDA^c^ (%)	KNN^d^ (%)	DT^e^ (%)	NB^f^ (%)	NN^g^ (%)
**Balanced**								
	1	2.5	100.0	100.0	100.0	100.0	100.0	100.0
	13	2.0	92.3	92.3	84.6	92.3	92.3	92.3
	316	1.5	90.2	88.2	80.4	80.4	89.2	88.3
	1884	1.0	75.6	74.9	68.2	68.8	68.4	77.0
	1321	0.5	70.5	70.6	63.5	65.9	65.4	71.1
	952	0.0	72.0	72.4	62.8	68.8	66.2	73.9
	1346	−0.5	70.9	70.6	60.4	67.1	63.7	72.1
	1955	−1.0	77.0	77.1	70.9	75.4	75.2	78.3
	288	−1.5	94.8	94.8	83.3	91.0	95.5	94.5
	3	−2.0	100.0	100.0	100.0	100.0	100.0	100.0
**Imbalanced**								
	1	2.5	100.0	100.0	100.0	100.0	100.0	100.0
	30	2.0	93.3	93.3	76.7	73.3	93.3	93.3
	571	1.5	77.4	75.7	64.1	71.1	77.4	78.3
	1886	1.0	70.6	70.3	63.9	65.0	64.6	73.3
	1537	0.5	76.3	75.5	67.3	71.2	72.7	79.7
	1251	0.0	78.7	78.0	75.6	74.5	76.8	80.3
	2794	−0.5	75.0	74.5	71.0	72.1	72.3	78.4
	2722	−1.0	88.3	88.3	85.0	83.3	87.1	89.1
	325	−1.5	99.1	99.1	96.6	98.2	99.1	98.8

^a^CDI: classification difficulty index.

^b^LR: logistic regression.

^c^LDA: linear discriminant analysis.

^d^KNN: K-nearest neighbor.

^e^DT: decision tree.

^f^NB: naive Bayes.

^g^NN: neural network.

**Table 8:** Electronic intensive care unit classification performance in traditional metrics.

Metric		LR^a^ (%)	LDA^b^ (%)	KNN^c^ (%)	DT^d^ (%)	NB^e^ (%)	NN^f^ (%)
**Balanced**							
	Accuracy	77.9	77.4	67.2	76.7	66.6	84.7
	Precision	77.9	78.1	67.9	76.7	73.7	84.5
	Recall	77.9	76.3	65.3	76.8	51.6	84.9
	F1	77.8	77.2	66.6	76.7	60.7	84.7
	AUC^g^	77.9	77.4	67.2	77.1	66.6	85.9
**Imbalanced**							
	Accuracy	78.0	80.1	73.6	81.6	73.3	89.5
	Precision	73.6	75.1	64.1	72.1	62.0	84.7
	Recall	62.1	60.2	47.2	72.9	51.5	83.5
	F1	67.4	66.8	54.4	72.5	56.3	84.1
	AUC	75.5	75.1	67.0	79.3	67.9	87.8

^a^LR: logistic regression.

^b^LDA: linear discriminant analysis.

^c^KNN: K-nearest neighbor.

^d^DT: decision tree.

^e^NB: naive Bayes.

^f^NN: neural network.

^g^AUC: area under the curve.

**Table 9:** Item response theory–based electronic intensive care unit mortality prediction accuracy stratified by classification difficulty index.

Number of cases		CDI^a^		LR^b^ (%)		LDA^c^ (%)		KNN^d^ (%)		DT^e^ (%)		NB^f^ (%)		NN^g^ (%)	
**Balanced**															
	2		3.0		100.0		100.0		100.0		50.0		100.0		100.0
	61		2.5		82.0		82.0		75.4		78.7		86.9		85.2
	160		2.0		81.3		82.5		75.0		76.3		81.9		83.4
	621		1.5		86.2		86.8		74.5		79.2		83.7		87.9
	3167		1.0		83.7		82.9		72.1		78.3		66.3		85.4
	4998		0.5		74.0		72.7		64.7		73.1		55.2		80.9
	4776		0.0		70.9		70.1		58.5		71.5		57.3		80.0
	3864		−0.5		73.8		74.5		63.3		74.4		67.4		84.3
	2858		−1.0		85.4		85.5		74.4		84.8		83.1		91.8
	1183		−1.5		92.5		92.6		84.3		91.7		91.9		96.4
	240		−2.0		97.1		97.1		91.7		95.8		96.3		97.9
	10		−2.5		100.0		100.0		100.0		100.0		100.0		100.0
**Imbalanced**															
	6		3.0		66.7		83.3		83.3		66.6		66.6		83.3
	58		2.5		82.8		81.0		69.0		75.9		87.9		84.5
	215		2.0		79.1		78.6		67.0		72.6		76.3		82.3
	1369		1.5		79.8		79.0		65.4		75.2		72.8		85.7
	4776		1.0		72.2		72.4		61.6		74.8		58.4		83.9
	6657		0.5		67.3		67.0		72.1		57.3		57.3		83.1
	7068		0.0		76.4		76.9		70.0		78.8		70.3		88.5
	6396		−0.5		87.1		87.3		83.2		87.3		83.4		93.7
	4265		−1.0		94.8		95.0		92.0		94.3		92.7		97.7
	1763		-1.5		98.0		98.0		97.1		97.9		97.3		99.4
	317		-2.0		99.1		99.1		98.4		98.4		98.4		99.1
	20		-2.5		100.0		100.0		100.0		100.0		100.0		100.0

^a^CDI: classification difficulty index.

^b^LR: logistic regression.

^c^LDA: linear discriminant analysis.

^d^KNN: K-nearest neighbor.

^e^DT: decision tree.

^f^NB: naive Bayes.

^g^NN: neural network.

### Effect Testing

The CDI group sizes at the extreme ends were too small and were collapsed into the next level down for each data set. Tests of the effects of MIMIC-III are reported in Table 10 and Figure 4.

The MIMIC-III balanced data showed significantly better accuracies for the more peripheral than central CDI bins. K-nearest neighbors and decision tree were the poorest classifiers. Although there was a small significant interaction effect, by and large, the main effects were borne out.

**Table 10:** Tests of the effects of classification difficulty index, classifier, and their interaction for the Medical Information Mart for Intensive Care III data set.

Effect		Significance		Significant paired comparisons (*P*<.001; higher accuracies listed first)
		*F* test (df)	*P* value	

**Balanced**				
	CDI^a^	123 (6,48456)	<.001	−1.5 vs −1.0, −0.5, 0.0 −1.0 vs −.05, 0.0 +1.0 vs +0.5, 0.0 +1.5 vs +1.0, +0.5, 0.0
	ML^b^ classifier	52 (5,48456)	<.001	LR^c^, LDA^d^, NB^e^, NN^f^ vs KNN^g^, DT^h^ DT vs KNN
	CDI×ML classifier	2 (30,48456)	<.001	−1.5: LR, LDA, NB, NN, DT vs KNN −1.0: LR, LDA, NB, NN, DT vs KNN −0.5: LR, LDA, DT, NN vs NB, KNN 0.0: LR, LDA, DT, NN vs NB, KNN +0.5: LR, LDA, NN vs NB, KNN, DT +1.0: LR, LDA, NN vs NB, KNN, DT +1.5: LR, LDA, NB, NN vs KNN DT
**Imbalanced**				
	CDI	314 (6,72660)	<.001	−1.5 vs −1.0, −0.5, 0.0 −1.0 vs −.05, 0.0 0.0 vs −0.5, +0.5, +1.0 +0.5 vs +1.0 +1.5 vs +1.0
	ML classifier	12 (5,72660)	<.001	LR, LDA, NB, NN vs KNN, DT
	CDI×ML classifier	2 (30,72660)	.004	−1.5: no differences −1.0: LR, LDA, NB, NN vs KNN, DT −0.5: LR, LDA, NN vs NB, KNN, DT 0.0: NN vs DT

^a^CDI: classification difficulty index.

^b^ML: machine learning.

^c^LR: logistic regression.

^d^LDA: linear discriminant analysis.

^e^NB: naive Bayes.

^f^NN: neural network.

^g^KNN: K-nearest neighbor.

^h^DT: decision tree.

The MIMIC-III imbalanced data set showed that at the healthier end of the CDI continuum, more peripheral cases were accurately classified. This was not the case at the central and unhealthier end of the continuum. Like the balanced data set, K-nearest neighbors and decision tree were the poorest classifiers. Although the interaction was significant, most of the paired comparisons supported the main effect findings.

Tests of the effects from eICU are reported in Table 11 and Figure 5.

**Table 11:** Tests of the effects of classification, classifier, and their interaction for the electronic intensive care unit data set.

Effect		Significance				Significant paired comparisons (*P*<.001; higher accuracies listed first)	
		*F* test (df)		*P* value			
**Balanced**							
	CDI^a^		382 (8,131586)		<.001		−2.0 vs −1.5, −1.0, −0.5, 0.0 −1.5 vs −1.0, −0.5, 0.0 −1.0 vs −.05, 0.0 +1.0 vs +0.5, 0.0 +1.5 vs +1.0, +0.5, 0.0 +2.0 vs +0.5, 0.0
	ML^b^ classifier		58 (5,131586)		<.001		NN^c^ vs LR^d^, LDA^e^, DT^f^ vs NB^g^ vs KNN^h^
	CDI×ML classifier		9 (40,131586)		<.001		−2.0: NN vs KNN −1.5: NN vs LR, LDA, NB, DT vs KNN −1.0: NN vs LR, LDA, NB, DT vs KNN −0.5: NN vs LR, LDA, DT vs NB vs KNN 0.0: NN vs LR, LDA, DT vs NB vs KNN +0.5: NN vs LR, LDA, DT vs KNN vs NB +1.0: NN vs LR, LDA vs DT vs KNN vs NB +1.5: NN, LR, LDA vs NB vs DT vs KNN −2.0: NN vs KNN
**Imbalanced**							
	Difficulty CDI		1138 (8,197406)		<.001		−2.0 vs −1.0, −0.5, 0.0 −1.5 vs −1.0, −0.5, 0.0 −1.0 vs −.05, 0.0 −0.5 vs 0.0 0.0 vs +0.5, +1.0 +1.0 vs +0.5 +1.5 vs +0.5, +1.0 +2.0 vs +1.0, +0.5
	ML classifier		28 (5,197406)		<.001		NN vs LR, LDA vs DT vs NB, KNN
	CDI×ML classifier		4 (40,197406)		<.001		−2.0: no differences −1.5: NN vs LR, LDA, NB, KNN, DT −1.0: NN vs LR, LDA, DT vs KNN, NB −0.5: NN vs LR, LDA, DT vs KNN, NB 0.0: NN vs LR, LDA vs DT vs KNN, NB +0.5: NN vs LR, LDA vs DT vs KNN, NB +1.0: NN vs LR, LDA, DT vs KNN, NB +1.5: NN, LR vs LDA vs DT, NB vs KNN +2.0: NN, LR vs KNN

^a^CDI: classification difficulty index.

^b^ML: machine learning.

^c^NN: neural network.

^d^LR: logistic regression.

^e^LDA: linear discriminant analysis.

^f^DT: decision tree.

^g^NB: naive Bayes.

^h^KNN: K-nearest neighbor.

For the eICU balanced data set, moving away from the central bin showed significantly better accuracy, except at the +2.0 level, which was similar to the +1.5 ML classifier estimated means showed that the neural network had significantly better accuracy than all other classifiers. The overall interaction effect was significant, but the paired comparisons were similar to the main effects.

For the eICU imbalanced data set, more peripheral cases were accurately classified at the healthier end of the distribution, whereas there was only a slight improvement at the unhealthier end. Similar to the other analyses, the neural network showed the best classification accuracy. Although the overall interaction was significant, the neural network continued to be the best classifier.

## Discussion

### Principal Findings

The results generally supported the hypothesis that cases with more extreme IRT-based CDI values are more likely to be correctly classified than cases with more central CDI values. This provides a unique manner to evaluate the utility of ML classifiers in a health context. We were able to demonstrate that ML classifiers performed similarly for the extreme cases, whereas for the centrally located cases, there were more differences between classifiers. Thus, ML classifiers can be evaluated based on their relative performance with cases of varying difficulty.

Although these were the general results, there were several specific findings that are worth noting. First, the neural network classifier was the best across all situations. The logistic regression and linear discriminant analysis classifiers were close to the second-best classifiers, whereas K-nearest neighbors almost always performed the worst. It is possible, as found in this study, that classifiers may turn out to be consistent over all levels of difficulty. However, owing to the unique characteristics of both data sets and classifiers selected, some algorithms may yield better results at various levels of case difficulty in other samples.

It was also clear that the *peripheral-central* trend of correct classification was most closely adhered to for cases with negative CDI values (ie, at the *healthier* end of the CDI distribution), and this trend was particularly pronounced with the imbalanced (2/3 *nondeath*) data sets. We adopted this modest imbalance in this research to detect trends such as these. This finding is pertinent to ML training protocols in that it is best to train them on balanced data sets before running them on imbalanced ones. There is a clear training effect toward negative CDI or the majority class in our case.

On the basis of the IRT analysis results, easier- and harder-to-classify cases were identified. This has implications for research and clinical practice. Once the cases have been identified, other information gathered from their patient-specific data may provide clues about why they are easier or harder to classify, diagnose, or treat. The features themselves that have varying weighted importance in the indexing process can be examined to assess for any differences in a patient’s CDI, that is, not just how many they got *wrong* but which they got *wrong* or *correct* to justify their position in eluding an ML classifier.

As an example of how one could examine more closely the *problematic* patients, we selected the neural network accuracies for each case in the *0* CDI bin in the MIMIC-III balanced data set. This provided 952 cases, 704 (73.9%) were correctly classified and 248 (26.1%) were not. A series of chi-square analyses were conducted using the *in and out-of-range* coding for each of the features crossed with accuracy. Not surprisingly, these cases did not differ on most of the features; the only ones with differences were WBC max (χ^2^_1_=5.6; *P*=.02), where those who were more accurately classified had out-of-range scores; bilirubin max (χ^2^_1_=4.2; *P*=.04), where those who were more accurately classified had normal scores; and mean heart rate (χ^2^_1_=7.1; *P*=.008, where those who were more accurately classified had normal scores. Using an approach like this can assist in determining which features in more problematic cases may be differentiated.

### Relationship With Previous Work

An IRT analysis can assist in providing a better understanding of why the classification process works well or falls short on the set of features and cases under investigation. This moves the field closer to having interpretable and explainable results [53,54]. Recent research with another ICU data set also argues about the importance of explainable processes as well as results [55]. Early research into ML focused on knowledge as an outcome and adopted an informal approach to evaluation. As the field has progressed, the focus shifted to large data sets, mathematical formulae, single evaluation metrics, and statistics, which has impoverished the discipline [22]. “Choosing performance metrics and confidence estimation methods blindly and applying them without any regard for their meaning and the conditions governing them, is not a particularly interesting endeavor and can result in dangerously misleading conclusions” [9].

### Limitations and Future Research

Limitations of this research include the fact that classifiers showcased here were not exhaustive, only ICU data sets were used, and converting an out-of-range laboratory value as either *in range=0* or *out of range=1* is reductive. Although this is true, the purpose of this study is to demonstrate a new evaluation metric using a basic 2PL model with binary data.

There are several ways to extend this work. Future research calls for (1) applying this method to other data sets to generalize its use, (2) using polytomous IRT models (eg, 0=in range, 1=somewhat out of range, and 2=very out of range) for more fine-grained case CDI scoring, (3) using multidimensional IRT models to obtain CDIs on >1 underlying dimension, and (4) using this approach to compare human versus machine classification accuracy across case difficulty. We can extend the intersection of ML with clinical medicine if we liken a physician to an ML classifier using feature data. It would be particularly interesting to compare case accuracies based on traditional ML versus clinical classifiers for cases of varying difficulty using an approach similar to that demonstrated in this study. Identifying which cases clinical classifiers are better suited to address, and which cases should be offloaded to an automated system allows for the optimal use of scarce resources. As clinical expertise is developed over time, the use of ML algorithms to assist any single individual would be a moving target and would also serve as a source of future research.

Another way to improve the veracity of the findings would be to address the issue of extraneous features. Several of the features in MIMIC-III and eICU had very low (<0.35) discrimination (slope) parameters, suggesting that there was a lot of *noise* in the cases’ CDIs as well as in the ML classifications. It would be a useful exercise to *a priori* determine the most useful features [5] and then run the analyses outlined in this study using a more refined feature set.

### Conclusions

As more ML methods are investigated in the health care sphere, concerns have risen because of a lack of understanding regarding why they are successful, especially when compared with physician counterparts. This study has suggested an IRT-based methodology as one way to address this issue by examining the case difficulty in a data set that allows for follow-up into possible reasons why cases are or are not classified correctly.

Using the methods described in this study would signal a change in the way we evaluate supervised ML. Adopting them would move the field toward more of an evaluation system that characterizes the entire data set on which the classifiers are being trained and tested. Doing so circumvents the pitfalls associated with 1 classifier being cited as more accurate or more precise and generates a more tailored approach to ML classifier comparisons. In addition, this methodology lends itself well to *post hoc* inspections of the data as to what makes difficult cases challenging.

The method here presents an intersection of personalized medicine and ML that maintains its explainability and transparency in both feature selection and modeled accuracy, both of which are pivotal to their uptake in the health sphere.

## Abbreviations

2PL: 2-parameter logistic
AUC: area under the curve
BUN: blood urea nitrogen
CDI: classification difficulty index
eICU: electronic intensive care unit
GLMM: generalized linear mixed model
ICU: intensive care unit
IRT: item response theory
MIMIC: Medical Information Mart for Intensive Care
ML: machine learning
